# Intraosseous pneumatocyst of the scapula: A case report

**DOI:** 10.1016/j.radcr.2024.07.029

**Published:** 2024-08-06

**Authors:** Jordan Tan Southi, Thomas Estephan, Amer Mitchelle, Robert Loneragan

**Affiliations:** Department of Radiology, Concord Repatriation and General Hospital, Sydney, Australia

**Keywords:** Pneumatocyst, Intraosseous, Scapula, Benign bone lesions, Computed tomography, Magnetic resonance imaging

## Abstract

Intraosseous pneumatocysts are benign, gas-filled cystic structures of bone, typically asymptomatic and discovered incidentally on imaging. Their precise aetiology remains unclear, with the prevailing hypothesis being that they result from air accumulation within the bone due to a vacuum phenomenon, typically linked to an adjacent joint space or intervertebral disc. We report the case of a 37-year-old man with an incidental intraosseous pneumatocyst of the scapula, which was evaluated with CT and MRI. Using thin-slice CT, we are able to detect a tiny cortical breach suggestive of a communication between the lesion and the adjacent glenohumeral joint, lending support to the aforementioned aetiological hypothesis.

## Introduction

Intraosseous pneumatocysts were first described in 1984 by Ramirez et al., with reference to localized gaseous locules within the subchondral iliac bone adjacent to the sacroiliac joint [1]. Case reports of the lesion have demonstrated a predilection for the ilium, sacrum, and vertebral bodies—particularly involving the lower cervical spine [2]. There are rare reports of intraosseous pneumatocysts within a cervical rib, the clavicle, scapula, head of humerus, pubis and acetabulum [[3], [4], [5], [6], [7], [8]]. Among these, the scapula is a notably rare site, described only once in the literature by Kamba et al. in 2000 [9]. To our knowledge, this is the first MRI evaluation of a scapula intraosseous pneumatocyst described in the literature.

## Case presentation

**Fig. 1:**
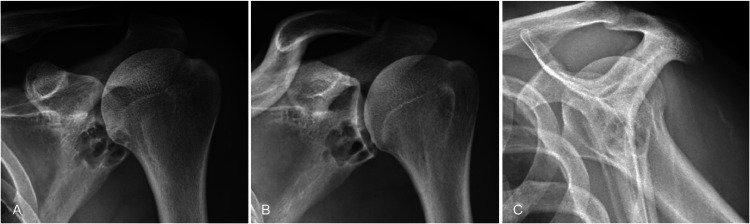
Left shoulder X-rays obtained in (A) anteroposterior, (B) Grashey, and (C) lateral views demonstrate the lucent lesion in the subchondral bone of the inferior glenoid.

A 37-year-old man with no comorbidities was admitted to the Intensive Care Unit for airway monitoring in the context of an acute respiratory illness. A chest X-ray on admission revealed an incidental rounded, multi-loculated, lucent lesion within the inferior aspect of the glenoid of the left shoulder. A dedicated X-ray of the left shoulder showed the lesion had well-demarcated margins, no periosteal reaction, pathological fracture or other high-grade features (Fig. 1). The patient was asymptomatic, and musculoskeletal examination of the left shoulder was unremarkable.

**Fig. 2:**
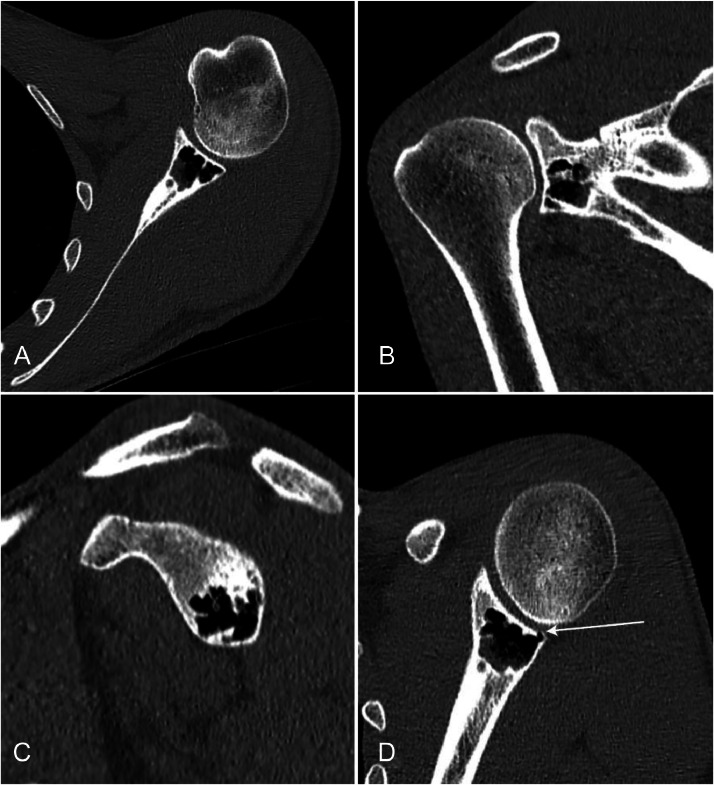
CT noncontrast of the left shoulder with (A) axial, (B) coronal, and (C) sagittal views demonstrates a well-defined lucent lesion within the subchondral bone at the level of the inferior glenoid. There are areas of gas density laterally (-875 HU) as well as scattered areas of fluid density medially (15 HU). An oblique view (d) was obtained via manual multiplanar reformatting, which highlights a tiny cortical breach posteroinferiorly (white arrow) suggestive of a communication between the lesion and the glenohumeral joint space. No aggressive features.

Noncontrast computed tomography (CT) was performed to characterize the lesion utilizing 0.625 mm axial slices (Fig. 2). This demonstrated a well-defined subchondral lucent lesion measuring 21 × 19 × 19 mm (axial x craniocaudal) at the inferior aspect of the glenoid. The lesion contained areas of gas density laterally (-875 Hounsfield Units [HU]) with scattered areas of fluid density medially (15 HU). A tiny cortical breach was noted at the posteroinferior glenoid rim, suggesting a communication between the lesion and adjacent glenohumeral joint space. CT again confirmed no aggressive features, including no pathological fracture and bland internal bone matrix.

**Fig. 3:**
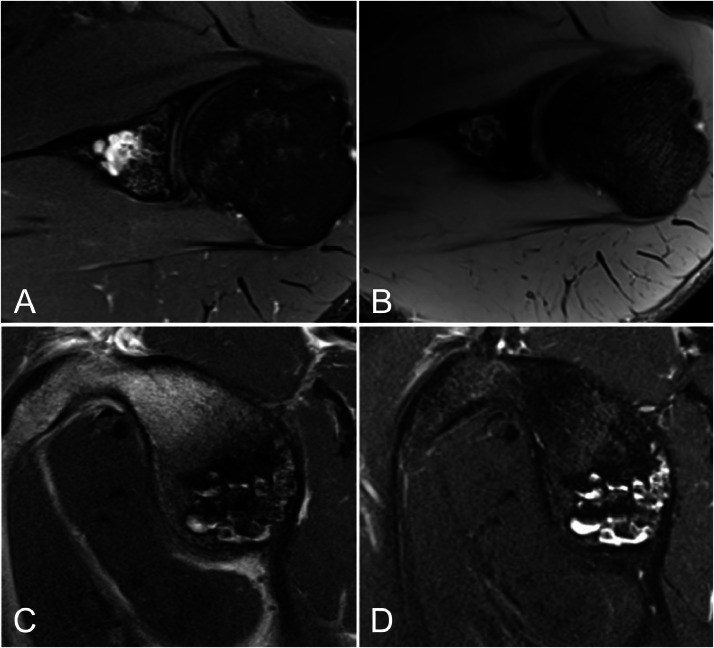
MRI of the left shoulder with (A) proton density fat-saturated axial, (B) T2-weighted gradient echo axial, (C) proton density sagittal, and (D) T2-weighted fat-saturated sagittal sequences. Multiple gas-fluid levels are demonstrated. There is no evidence of intra-lesional fat.

**Fig. 4:**
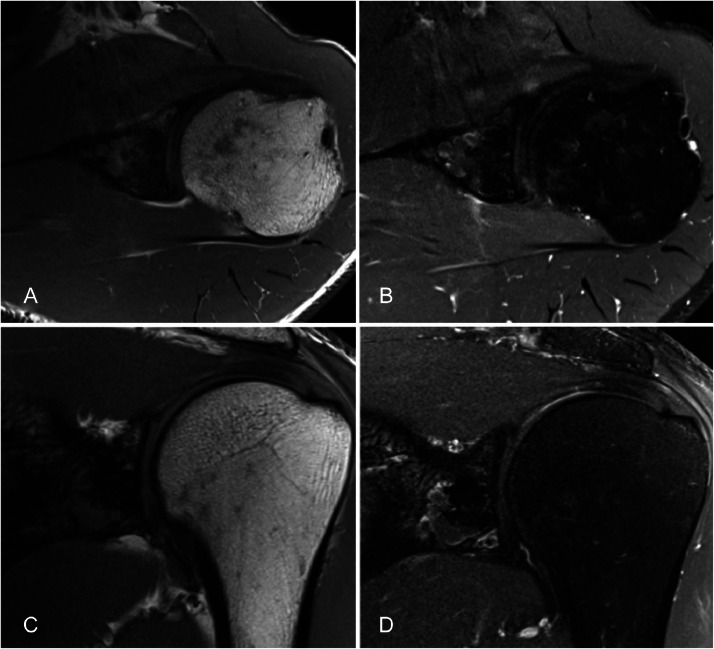
MRI of the left shoulder with (A) T1-weighted axial, (B) T1-weighted fat-saturated post gadolinium, (C) T1-weighted coronal, and (D) T1-weighted fat-saturated post gadolinium coronal sequences. No enhancing component is demonstrated within the lesion.

Magnetic resonance imaging (MRI) was performed using a 3T MRI machine (Fig. 3). The lesion demonstrated multiple well-defined high T2 intensity foci with fluid-fluid levels. There was no evidence of intra-lesional fat. Multiple foci of blooming artefact along the lateral aspect of the lesion correspond to the gas locules demonstrated on CT. T1 and T1-fat saturated postgadolinium sequences demonstrated no enhancing component (Fig. 4).

## Discussion

A systematic review published in 2013 by Oehler et al. reviewing 152 previously published cases of intraosseous pneumatocysts demonstrated a predilection towards patients that are male and over the age of 50 [3]. Although previously described as rare, numerous studies have conversely demonstrated that these lesions may be more common than initially thought. Prassapoulos et al. carried out a prospective study in 1999 which examined 369 pelvic CT scans and reported a prevalence of sacroiliac joint pneumatocysts of 10.8% [10]. Meanwhile, a retrospective study published by Matsukubo et al. of 500 cervical spine CT scans in 2013 yielded an overall prevalence of intravertebral pneumatocysts of 42% [11]. The higher observation rate of intraosseous pneumatocysts in Matsukubo's study may be attributed to the utilization of thinner slice images (1 mm), as opposed to the 2-3 mm slices used in previous studies. This allows reduction of partial volume artifacts that might have hindered the detection of pneumatocysts.

The pathogenesis of intraosseous pneumatocysts is of debate, with various competing hypotheses proposed in the literature. Nonetheless, there is a consensus that these lesions are acquired in nature, as evidenced by several studies which examined X-rays taken prior to their detection [3]. Initially, Ramirez et al. noted the absence of any discernible communication with the adjacent joint space. It was therefore proposed that that they form due to either the spontaneous accumulation of intraosseous air, or the secondary accumulation of gas within a simple intraosseous fluid-filled cyst [1]. In contrast, the prevailing theory now suggests that intraosseous pneumatocysts result from the migration of gas from nearby joint spaces or intervertebral discs into the bone through a vacuum phenomenon [2,3]. This concept is supported by their tendency to be juxta-articular and their correlation with adjacent degenerative lesions and advanced age [3]. Furthermore, Matsukubo et al.’s study demonstrated a higher incidence of joint space communication compared to other studies via the use of thin 1 mm axial computed tomography slices, providing indirect evidence that intraosseous air has an external source [11].

Similarly, our case uses 0.625 mm axial slices, and is thus able to demonstrate a tiny cortical breach of the pneumatocyst margin. This is suggestive of communication with the adjacent glenohumeral joint. In contrast, Kamba et al.'s case of a scapular pneumatocyst was unable to identify evidence of a communication via the use of 3 mm slices [4]. It is suggested that microscopic communications with the joint space may be radiographically occult on CT images, particularly in the context of thicker slices [12].

Notably our patient was a healthy 37-year-old male, with no features of degenerative arthropathy at the glenohumeral joint. There are several accounts of intraosseous pneumatocysts occurring in similarly noncomorbid individuals who documented intravertebral pneumatocysts in younger patients aged between 33 to 45 years without degenerative changes [2,3,13]. This suggests an alternative mechanism separate from degenerative disease, perhaps from remote trauma or from a congenital abnormality as proposed by Siddiqui et al. [14].

The natural history of intraosseous pneumatocysts is variable. However, its benign nature has been confirmed in the previous literature, via biopsy in some cases. These demonstrate a nonspecific fibrous capsule of mesenchymal origin [1,3,15]. While the majority of these lesions have been shown to remain static in size over time, there have been 3 documented occurrences of enlargement, all within the vertebrae [[16], [17], [18]]. Notably, Dash et al. report a case of an enlarging C5 pneumatocyst causing spinal cord compression and myelopathy, requiring C5 corpectomy [16].

The fluid levels within the intraosseous pneumatocyst in this case have been described previously. The lesions have been described to transition from air filled cystic lesions to fluid filled lesions within the span of months, with 1 case illustrating eventual filling with granulation tissue over 40 weeks [16,17]. The mechanism for fluid accumulation is unclear and has been proposed to be due to a pressure differential between the pneumatocyst cavity and the surrounding bone marrow [16]. Conversely, supporting Ramirez's original pathogenic hypothesis for these lesions, other authors have suggested that the fluid within intraosseous pneumatocysts might originate from a pre-existing simple fluid-filled cyst, ganglion, or synovial cyst that has involuted [19].

In conclusion, we describe an incidental intraosseous pneumatocyst of the inferior subchondral glenoid in an otherwise healthy 37-year-old man. Using thin 0.625 mm axial CT slices, we are able to detect a tiny cortical breach posteroinferiorly suggesting a communication with the glenohumeral joint, lending support to the theory that the gas of intraosseous pneumatocysts originates from the adjacent joint space or intervertebral disc. These lesions exhibit a characteristic appearance on CT, featuring well-defined rounded areas with air attenuation, and may also include fluid components [3]. Familiarity with these benign lesions is important for clinicians and radiologists to not mistake these for sinister pathologies such as emphysematous osteomyelitis, osteonecrosis, or malignancy and thereby avoid unnecessary investigations and patient distress.

## Patient consent

Complete written informed consent was obtained from the patient for the publication of this case report.
